# Do Non-Decision Times Mediate the Association between Age and Intelligence across Different Content and Process Domains?

**DOI:** 10.3390/jintelligence8030033

**Published:** 2020-09-01

**Authors:** Mischa von Krause, Veronika Lerche, Anna-Lena Schubert, Andreas Voss

**Affiliations:** Department of Psychology, Heidelberg University, 69117 Heidelberg, Germany; veronika.lerche@psychologie.uni-heidelberg.de (V.L.); anna-lena.schubert@psychologie.uni-heidelberg.de (A.-L.S.); Andreas.Voss@psychologie.uni-heidelberg.de (A.V.)

**Keywords:** diffusion modeling, cognitive aging, response time, intelligence

## Abstract

In comparison to young adults, middle-aged and old people show lower scores in intelligence tests and slower response times in elementary cognitive tasks. Whether these well-documented findings can both be attributed to a general cognitive slow-down across the life-span has become subject to debate in the last years. The drift diffusion model can disentangle three main process components of binary decisions, namely the speed of information processing, the conservatism of the decision criterion and the non-decision time (i.e., time needed for processes such as encoding and motor response execution). All three components provide possible explanations for the association between response times and age. We present data from a broad study using 18 different response time tasks from three different content domains (figural, numeric, verbal). Our sample included people between 18 to 62 years of age, thus allowing us to study age differences across young-adulthood and mid-adulthood. Older adults generally showed longer non-decision times and more conservative decision criteria. For speed of information processing, we found a more complex pattern that differed between tasks. We estimated mediation models to investigate whether age differences in diffusion model parameters account for the negative relation between age and intelligence, across different intelligence process domains (processing capacity, memory, psychometric speed) and different intelligence content domains (figural, numeric, verbal). In most cases, age differences in intelligence were accounted for by age differences in non-decision time. Content domain-general, but not content domain-specific aspects of non-decision time were related to age. We discuss the implications of these findings on how cognitive decline and age differences in mental speed might be related.

## 1. Introduction

Most cognitive abilities decline across the life-span (Hartshorne and Germine 2015; Salthouse 2004, 2010). This well-established finding holds true across a variety of process domains (e.g., general intelligence, reasoning, memory) and across different content domains (e.g., figural, numeric, verbal). Only for so-called crystallized abilities (Cattell 1963), which are largely knowledge-based, ability scores increase until people are in their sixties (Horn and Cattell 1967). One clear-cut result, found in cross-sectional as well as longitudinal data, is that older adults show slower response times than younger people in elementary cognitive tasks—a pattern that already starts in mid-adulthood (Hartshorne and Germine 2015; Jensen 2006; Salthouse 2010; Schaie 2005). As response times are linked to intelligence (Sheppard and Vernon 2008), it has been proposed that these age differences in response times might form the basis for the decline of a wide range of cognitive abilities-cognitive decline in general might be based on a slow-down of basic cognitive processes (Finkel et al. 2007; Salthouse 1996; Verhaeghen and Salthouse 1997). In fact, response times have been found to mediate the relationship between age and intelligence, lending support to the assumption that differences in cognitive speed might be responsible for age differences in intelligence (although findings in longitudinal studies show that the correlation between age-related change in processing speed and age-related change in intelligence is lower than the cross-sectional data suggest, see Lindenberger et al. 2011; Zimprich and Martin 2002).

Salthouse (1996) proposed that an age-related slow-down might affect cognition in two ways. First, because the time available for problem solving is typically limited, less time is available for higher-order information integration if the basic processes in early stages of information processing take too long. Second, based on the idea that information stored in working memory deteriorates over time, a slow-down in early processes might lead to greater losses of information before integration starts. Both accounts assume that processing speed reflects a general component of information processing that generalizes across content domains and task types. Thus, the same base might be responsible for all kinds of cognitive decline, across process domains and content domains. If that is the case, different aspects of cognitive ability should show correlated change. Findings in support of this notion of a general decline have been reported in the literature, although there is also evidence for domain-specific and task-specific aspects (Salthouse 2004; Sliwinski and Hall 1998; Tucker-Drob 2011).

Response times measured in elementary cognitive tasks are a widely used and long-established instrument to assess cognitive speed (Jensen 2006). However, the use of response times as a single indicator leads to at least two problems, both of which are related to the fact that response times are not a process-pure measure of cognitive speed. First, there can be a trade-off between speed and accuracy: Some people might try to respond as quickly as possible at the risk of making more mistakes, whereas others might be more inclined to be as accurate as possible, even if this leads to slower responses. Second, the time needed for sensory encoding and for motoric response execution is intermingled with the time needed for information processing. Thus, mean response times (and response time variances, too) are influenced by several processes that might not actually reflect processing speed.

**Figure 1:**
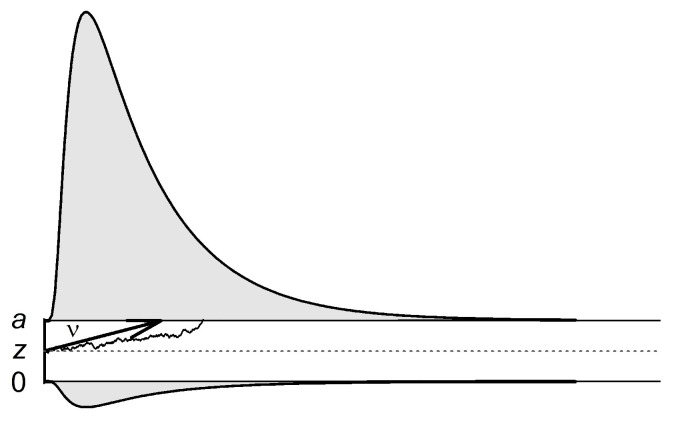
The diffusion model. The accumulation process starts at starting point *z*, moves with average slope *v*, and terminates when one of the two thresholds (0 or *a*) has been reached.

To gain more process-pure measures, the diffusion model (Ratcliff 1978; Ratcliff and McKoon 2008; Voss et al. 2013) can be applied—a stochastic model that takes into account both accuracy rates and response time distributions from binary decision tasks. Figure 1 shows a graphical representation of the model. The decision process is described as an evidence accumulation process with constant drift and random noise, starting at the point *z* between two decision boundaries. A decision is made, and motor response execution starts, as soon as the evidence accumulation process reaches one of the boundaries. One main advantage of the diffusion model is that it allows for disentangle (a) speed-accuracy trade-offs, (b) the speed of information processing, and (c) non-decisional components of response times. Among others, the model yields estimates of three parameters that reflect (a) the conservatism of the decision criterion (i.e., boundary separation *a*), (b) the speed of information processing or the quality of the evidence entering the decision process (i.e., drift rate *v*), and (c) the time needed for encoding and motor response execution (i.e., non-decision time *t*_0_). Experimental studies have demonstrated that these diffusion model parameters are valid measures of the respective components of the decision process (Arnold et al. 2015; Lerche et al. 2017; Voss et al. 2004).

The diffusion model thus provides parameter estimates that allow a model-based scrutiny of why older people’s response are slower. Are elder persons more careful in selecting the correct answer, focusing less on speed? Are they slower in their speed of information accumulation? Or do they take longer for encoding and motor response execution? Or does age-related slowing reflect a combination of these processes? The answers to these questions hint at different interpretations of what underlies the correlation between age differences in response times and age differences in cognitive abilities.

There is a growing number of studies on age differences in diffusion model parameters (Ball and Aschenbrenner 2018; Janczyk et al. 2018; Madden et al. 2010; McGovern et al. 2018; McKoon and Ratcliff 2012, 2013; Ratcliff 2008; Ratcliff et al. 2001, 2004, 2006, 2010; Spaniol et al. 2006, 2011; Thapar et al. 2003; Voskuilen et al. 2018). Dully et al. (2018) gave an overview of the state of the literature in their systematic review. They found consistent and robust age effects for boundary separation *a* and non-decision time *t*_0_. This suggests that elder people respond slower because they employ more conservative decision criteria and need more time for extra-decisional processes. In contrast to these clear-cut findings, age differences in drift rate vary notably across studies. This finding is surprising as it implies that there might be no general age-related slow-down in information processing. Age differences in response times might arise primarily or even exclusively due to the fact that older people are more careful and take longer for encoding and motor processes. In a recent meta-analysis of age differences in diffusion model parameters summarizing 25 samples, Theisen et al. (2020) studied task type as potential moderator of the link between age and drift rate. The authors found small negative age effects on drift rate in memory and simple perception tasks, but small positive age effects for drift rate in lexical decision tasks. The latter might be explained by the fact that performance in lexical decision tasks is partly based on vocabulary knowledge, an aspect of cognition that has been found to peak later in life than most other cognitive abilities, showing increases at least until the age of 50 (Hartshorne and Germine 2015; Horn and Cattell 1967; Salthouse 2004). Theisen et al. (2020) further examined task difficulty as a potential moderator. The meta-analysis suggested that in perceptual and lexical decision tasks older adults profited from increased task difficulty. However, for the moderator analyses, the number of available data sets was rather low so that these results should be interpreted with caution. Nevertheless, the findings from this meta-analysis suggest that age effects in drift rate might be highly dependent on the type of task (e.g., its domain and difficulty). An important limitation of most previous studies on age differences in diffusion model parameters is that they used only very few different tasks, typically only one (Spieler 2001). Thus, it remains an open question whether the effects found in different studies for different tasks are comparable.

Extending this argument, it should be noted that the studies examined in the meta-analysis all employed tasks with relatively short latencies and thus a restricted variance in complexity. In the past, most tasks analyzed with the diffusion model had mean response times of less than 1.5 s. However, recently, it has been demonstrated that the diffusion model also provides a good fit and valid results for more complex tasks with mean response times that are notably above 1.5 s (Lerche et al. 2018; Lerche and Voss 2019). In the present study, we will draw upon these findings and analyze the cognitive processes underlying age-related slowing based on a much larger variation of task complexity. Furthermore, previous diffusion model studies (e.g., McKoon and Ratcliff 2012; Ratcliff 2008; Ratcliff et al. 2001, 2004, 2010) typically used a group design, comparing young adults (i.e., college age) to old adults with a mean age above 60. It is an open question whether these results are generalizable to other age classes that is whether there are linear age trends for the model parameters across the whole span of adulthood. In our study, we focus on differences across young- and mid-adulthood, employing a continuous measure of age.

After establishing that there are systematic effects of age on the diffusion model parameters, the next step is to assess the role of these effects in age related differences in outcome measures like intelligence. Do changes in diffusion model parameters mediate the link between age and intelligence in the same way as mean response times do? Recently, Schubert et al. (2020) reported the first answers to this question. Using two different response time tasks, they found that non-decision time and latencies in event-related potentials (ERP) in the P3 component of the electroencephalogram (EEG) mediated the relationship between age and IQ as measured in a standard intelligence test. In contrast, age did not mediate the correlation between non-decision time and IQ, implying that the link between non-decision time and intelligence is not due to a confounding between age and non-decision time. The model parameter non-decision time is thought to reflect the time needed for encoding processes and motor response execution. As the authors did not find non-decision time to be related to early ERP latencies that might reflect encoding (i.e., N1 and P1), they proposed two possible (contrasting) explanations for the observed mediation effect of non-decision time: First, differences in non-decision time might reflect age-related differences in anterior brain regions that are associated with motor planning and response execution. Importantly, the same anterior brain regions might also affect latencies of ERP components occurring later in the stream of information-processing such as the P3 that are closely related to higher-order processing and intelligence (Schubert and Frischkorn 2020; Schubert et al. 2017). Second, the mediation via non-decision time might reflect the influence of non-decisional processes on the intelligence test scores because the test used (Berlin Intelligence Structure Test; Jäger et al. 1997) has strict time limits for each task and scores are thus affected by the speed of motor response execution (i.e., hand-writing).

One limitation of the results reported by Schubert et al. (2020) is the low number of response time tasks that were used in their study. The authors applied the Sternberg memory task and the Posner letter matching task, two well established paradigms. However, based on solely these two tasks, Schubert et al. (2020) could not examine influences of different intelligence components, content domains, or task complexities. It thus remains an open question whether the results of the mediation analyses hold (a) across different response time tasks from different content domains and from different complexity and (b) across different aspects and domains of intelligence. Both varieties should be studied using one and the same sample, to ensure full comparability and offer a clear picture of the relations between age, the diffusion model parameters, and intelligence. This paper aims precisely at closing this gap.

### The Present Study

The present study reanalyzes data from a large study on the structure of cognitive speed (Lerche et al. 2020). In the original publication, no age effects are reported. The study uses 18 response time tasks from three different content domains (figural, numeric, verbal). Half of the tasks are fast tasks (with mean RTs below 1 s) and half are more complex (mean RTs > 2 s). We tested a sample of adults with an age range of 18 to 62 years, thus spanning all of young- and mid-adulthood, as well as the beginning of (young) old adulthood. To investigate whether response times and diffusion model parameters mediate age differences in a range of cognitive abilities, we used the same intelligence test as Schubert et al. (2020) to obtain a score of *g*, but we also computed scores for three intelligence process domains (processing capacity, psychometric speed, and memory), and three intelligence content domains (figural, numeric, and verbal). For each of these intelligence scores, we analyzed whether the diffusion model parameters, aggregated across tasks, account for age effects. We expected to find positive age correlations for boundary separation and non-decision time, indicating that older adults use more conservative decision criteria and take longer for encoding and motor response execution processes. We did not expect to find any age correlations for drift rates, except for the verbal domain, where—according to the results from the meta-analysis of Theisen et al. (2020)—older adults might have an advantage because verbal abilities involve a strong element of knowledge that might increase across a large part of adulthood. We had no specific hypotheses about the impact of task complexity. Following the results of Schubert et al. (2020), we expected age effects in the intelligence scores to be mediated by non-decision time. We also tested the other main diffusion model parameters (threshold separation and drift rate) as possible mediators, as well as mean logarithmized response times. In addition, differentiating between the process domains allowed us to compare the different explanations of the non-decision time mediation offered by Schubert et al. (2020). If age-related differences in non-decision time reflect age-related changes in anterior brain regions linked to both response preparation and higher-order processes such as intelligence, the mediation should occur equally across process domains. However, if the mediation is based on the fact that the intelligence test tasks have strict time limits, the mediation via *t*_0_ should be especially strong for the psychometric speed intelligence tasks, and be less pronounced for the processing capacity intelligence tasks, which have more lenient time limits and are therefore less based on quick response execution and more similar to a power test.

## 2. Materials and Methods

Analyses based on the data of this study have also been reported by Lerche et al. (2020). The authors examined relationships between diffusion model parameters and intelligence and found both domain-general and domain-specific relationships between drift rate and intelligence. Age effects were not analyzed in their paper. Next, we will report the main aspects regarding sample characteristics, procedure, and material of the study. More details can be found in Lerche et al. (2020).

### 2.1. Participants

We determined our sample size based on a power analysis for structural equation model analyses reported in Lerche et al. (2020). We had a sample of 125 participants, leading to a power of 0.81 to detect correlations of *r* = 0.25 (α = 0.05). We recruited participants by means of a newspaper article, via fliers distributed at public places and by means of an online participant pool. All participants provided informed consent and received 35 € as well as feedback on their performance after completing the study. Our final sample (see below for a description of the proportion of missing data) consisted of 123 participants. The proportion of women amounted to 62.60% and 50.41% were students. The mean age was 35.85 years (SD=14.13), with a range of 18 to 62 years. 59 participants were 18–29 years old, 15 were 30–39 years old, 19 were 40–49 years old, and 30 were 50–62 years old, with five of them being 60 or older.

### 2.2. Procedure

Participants completed three data collection sessions. In session 1, participants filled in the BIS intelligence test (see below), while in sessions 2 and 3, they worked on response time tasks (nine in each session). The order of the tasks was identical across participants. Table 1 gives on overview of the RT tasks and their order in the study. In each response time task session, participants took a three-minute break after the third and sixth task.

In all RT tasks, people started with four practice trials with feedback on the correctness of their responses (green checkmark vs. red cross shown for 1.5 s), followed by one warmup trial and 100 test trials.

### 2.3. Material

#### 2.3.1. Intelligence Assessment

As a measure of intelligence, we used the Berlin Intelligence Structure Test (BIS; Jäger et al. 1997) that is based on the bimodal intelligence structure model (Jäger 1982). The test provides tasks for three different content domains (figural, numeric, verbal) and four different process domains (processing capacity, psychometric speed, memory, and idea fluency). We used a short version of the test and disregarded the three idea fluency tasks, leading to a final set of 12 intelligence test tasks. We excluded the idea fluency tasks in the current analyses as we were not interested in creativity. Four tasks stemmed from each of the content domains. The processing capacity scale consisted of six tasks (two from each content domain), while psychometric speed and memory were both measured with one task from each content domain. We computed scale means for general intelligence *g* (including all tasks used), the four process domains, and the three content domains. Please note that the BIS manual only gives scoring rules for processing capacity and *g* when the short version of the test is used—we computed the scale means for the other scales correspondingly. For three participants, we could not use the scores from two tasks due to disturbances during data collection.

#### 2.3.2. RT Tasks

We used three fast tasks (mean RT ca. 800 ms) and three slow and more complex tasks (mean RT ca. 3000 ms) for each of the three content domains (figural, numeric, verbal), leading to a total of 18 RT tasks (see Table 1).

In the fast figural tasks, people had to determine whether a dot was within or outside of a rectangle (FF1, dot-rectangle task), which of two rectangles shown on the left and right side of the screen covered the greater area (FF2, simple area task), and whether a polygon shown was a triangle or a rectangle (FF3, polygon task). Among the slow figural tasks was a maze task (SF1), where participants had to judge whether a way out of the maze could be found from a marked spot. Another slow figural task was an extended version of the simple area task: Participants now had to judge whether three rectangles marked in blue or three rectangles marked in red covered the greater total area (SF2, complex area task). Finally, in the pie task (SF3), people judged whether three “slices” of a pie plot added up to less or more than a total pie.

In the fast numeric tasks, people had to determine whether a number was greater or smaller than 500 (FN1, number discrimination task), whether it was odd or even (FN2, odd-even task), or whether a number shown on the left side of the screen was larger than a number on the right side (FN3, simple inequation task). Among the slow numeric tasks was the mean value computation task (SN1) where people had to determine whether the mean of 16 numbers was greater or smaller than 500. In the equation task (SN2), participants judged whether equations were correct or wrong (e.g., 5*7 = 25). Finally, in the complex inequation task (SN3), people had to decide whether the solution of an equation shown on the left side of the screen was larger than the solution of an equation shown on the right side (e.g., “9–6” vs. “19–17”).

In the fast verbal tasks, people judged whether a word was an adjective or noun (FV1, word category task), whether a letter combination was a word or not (FV2, lexical decision task), and whether a noun denoted a living versus a non-living entity (FV3, animacy task). Among the slow verbal tasks was a grammar task (SV1). People had to decide if the grammatical error in a five-word sentence was in the possessive pronoun or in the noun. In the statement task (SV2), in each trial, we presented four to six words scattered across the screen. People’s task was to determine whether a true statement could be formed from these words. Finally, in the semantic category task (SV3), people saw a list of five nouns (e.g., chair, sun, armchair, sofa, bench). People had to decide whether one or two of the items belonged to a different semantic category than the others. In the example, one of the nouns, i.e., “sun”, differs from the dominant category (i.e., furniture).

A more detailed description of all tasks is provided by Lerche et al. (2020).

### 2.4. Data Preparation

For all RT tasks, we excluded data from trials faster than 300 ms. In a second step, we also excluded intra-individual outliers, separately for each participant and each task. We defined outliers as RTs more than three interquartile ranges (IQRs) above the third quartile or three IQRs below the first quartile of the intra-individual RT distribution (Tukey 1977). One participant accidentally skipped two tasks, introducing some missing response time data. We removed diffusion model parameters from model estimations that resulted in an inadequate fit according to a simulation study, separately for each participant and task (for a description of the simulation study, see Lerche et al. 2020; 0.93% of data excluded). In the next step, we excluded the data separately for each task and participant, if the mean RT or accuracy rate were more than three IQRs away from the first or third quartile for this task. Finally, we excluded two participants as multivariate outliers because their Mahalanobis distance, based on all diffusion model parameter estimates, mean RTs, and the intelligence content domain scores, exceeded the critical value of $\chi^{2}=140.89\;(df=93,\;p=0.001$). The resulting sample thus contained 123 people.

### 2.5. Parameter Estimation

We used the maximum likelihood estimation procedure provided of fast-dm (Voss and Voss 2007, 2008; Voss et al. 2015) for obtaining estimates of diffusion model parameters. Simulation studies show that this procedure provides reliable parameter estimates for 100 trials (Lerche et al. 2017). We estimated parameters separately for each participant and each task. We used a simple model, estimating drift rate (*v*), boundary separation (*a*), non-decision time (*t*_0_), and the inter-trial variability of non-decision time (*st*_0_). The starting point (*z*) was fixed at the center between the two boundaries, as we associated the boundaries with correct and erroneous responses and thus did not except an a priori bias. We fixed all other parameters to zero, following recommendations by Lerche and Voss (2016). Across all tasks, model fit was good according to a graphical analysis and a simulation study (see Lerche et al. 2020, for a detailed description of model fit).

### 2.6. Data Analysis

We used *R* (R Core Team 2020) and the R-packages *papaja* (Aust and Barth 2018), *psych* (Revelle 2018), *scales* (Wickham and Seidel 2019), *lavaan* (Rosseel 2012), and *tidyverse* (Wickham et al. 2019) for all analyses. All data and the analysis script are available on the Open Science Framework (https://osf.io/xpbwe).

In a first step, we examined the bivariate correlations with age and general intelligence (*g*) for the diffusion model parameters, response times, and accuracy rates, separately for each task. In the next step, we computed the means of the z-standardized values across all tasks and separately for each content domain (for *v*, *a*, *t*_0_, and mean log RT). Our sample size was too small to fit a structural equation model including the three diffusion model parameters for all 18 tasks. Hence, we used scale means for the mediation analyses. Additionally, we also examined the task-specific age correlations. Cronbachs’s alpha values across all 18 tasks were good for threshold separation (α = 0.86) and acceptable for drift rate (α = 0.76) and non-decision time (α = 0.71).

#### Mediation Models

**Figure 2:**
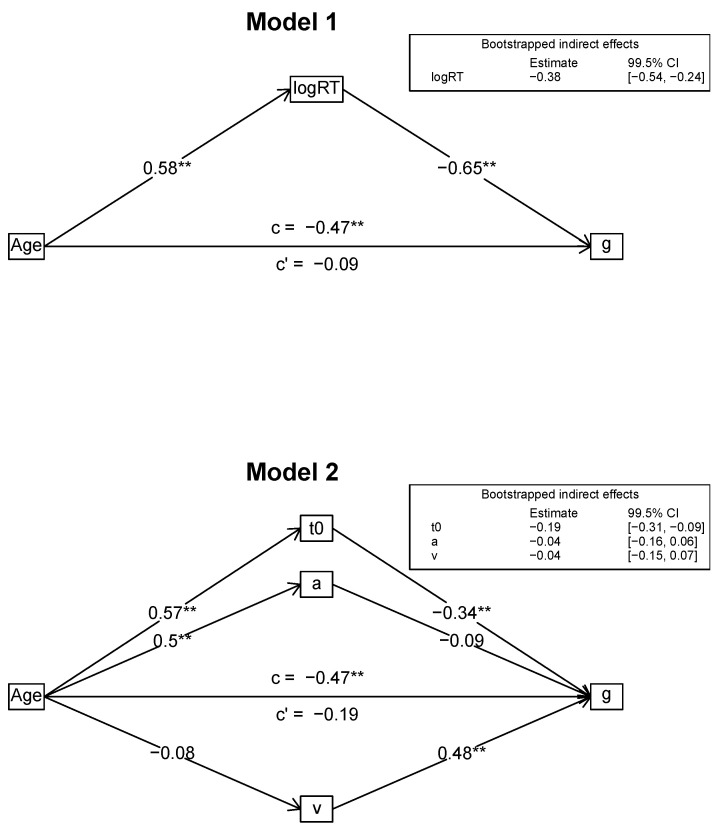
Mediation models for general intelligence. Standardized estimates are reported. ** *p* < 0.001.

**Figure 3:**
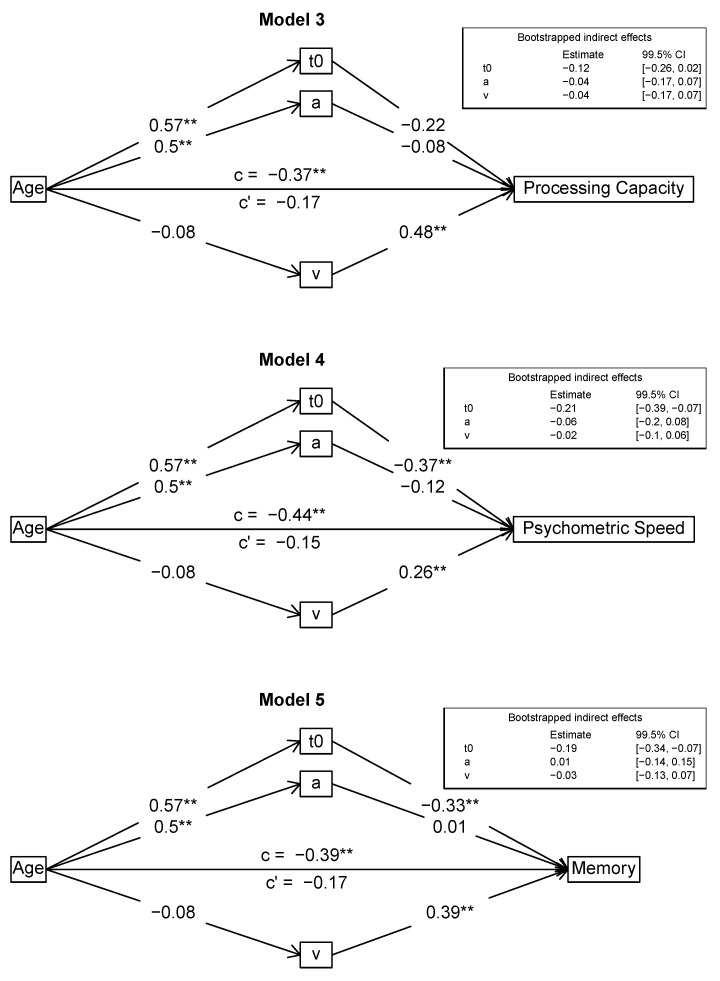
Mediation models for intelligence process domains. Standardized estimates are reported. ** *p* < 0.001.

**Figure 4:**
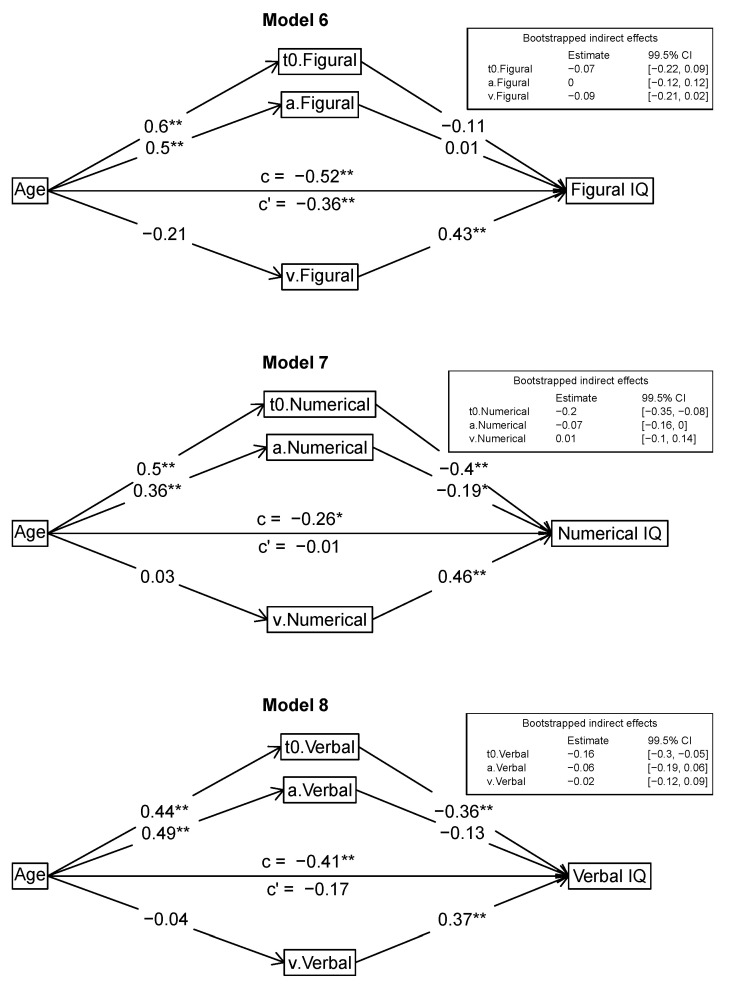
Mediation models for content domains. Standardized estimates are reported. * *p* < 0.005, ** *p* < 0.001.

We formulated and tested several different mediation models to examine the interplay between age, intelligence, and the diffusion model parameters in depth. For the mediation analyses, we used the *R* package *psych* that provides bootstrapped confidence intervals for the indirect effects. Specifically, we analyzed whether the diffusion model parameters can account for the age effects on different intelligence measures, that is *g*, the scores of the three different process domains (processing capacity, psychometric speed, and memory), and the three content domains (figural, numeric, verbal). Accordingly, in all models, age was the primary predictor variable. In the first two models, the outcome variable was *g*. As we wanted to first confirm the established finding that mean response times mediate the age/intelligence relation, we used mean logarithmized RTs as a mediator in Model 1. Then, the three diffusion model parameters (*v*, *a*, *t*_0_) served as mediators in Model 2, testing the assumption that these parameters can jointly account for the age-intelligence associations. In the next step, we tested mediation models for each of the process domains (processing capacity: Model 3; psychometric speed: Model 4; memory: Model 5), also using the diffusion model parameters as mediators. Finally, we examined whether the mediation was robust across content domains. We used content-domain specific diffusion model parameters (figural, numeric, verbal) as mediators of the relation of age to the respective intelligence domain scores (figural: Model 6; numeric: Model 7; verbal: Model 8). Model figures are given below (Figure 2, Figure 3 and Figure 4).

For all significance tests, we used a strict alpha level of α = 0.005 to account for multiple testing.

## 3. Results

### 3.1. Descriptive Statistics and Simple Correlations

**Figure 5:**
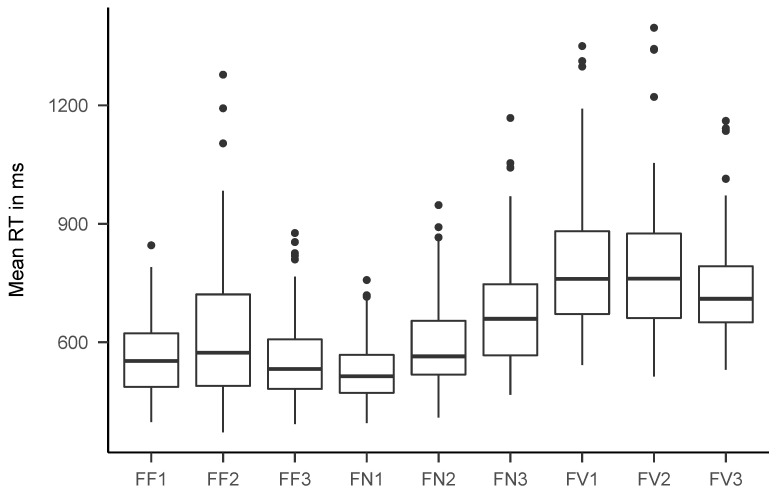
Boxplots of mean response times for all fast tasks. See Table 1 for an explanation of the task names.

**Figure 6:**
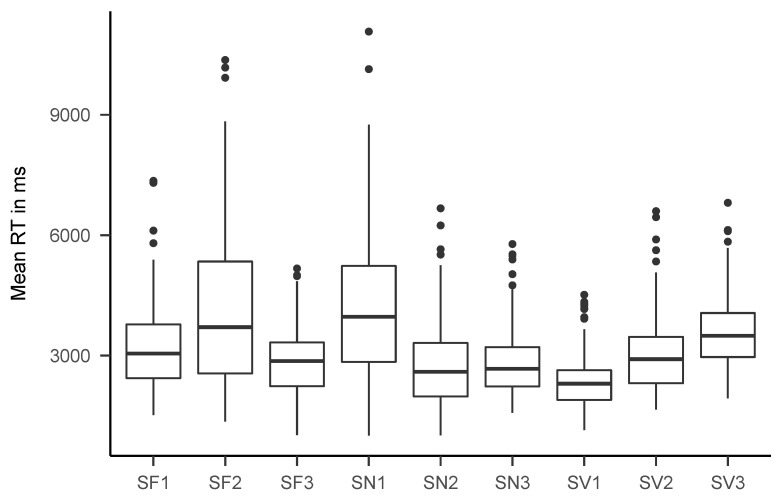
Boxplots of mean response times for all slow tasks. See Table 1 for an explanation of the task names.

**Figure A1:**
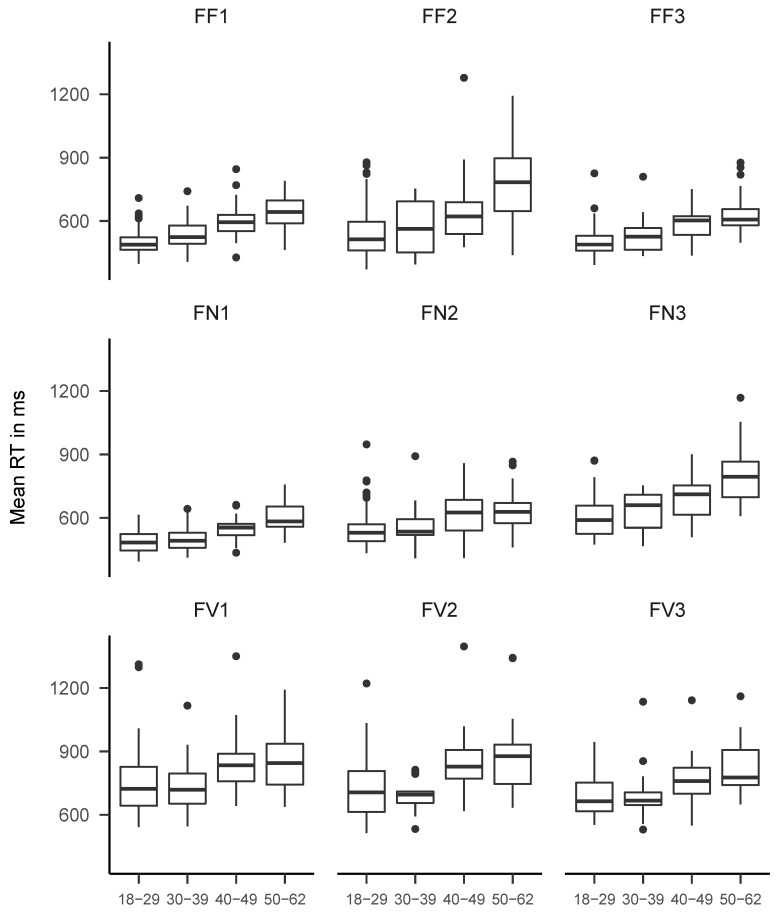
Boxplots of mean response times for all fast tasks, split by age groups. See Table 1 for an explanation of the task names.

**Figure A2:**
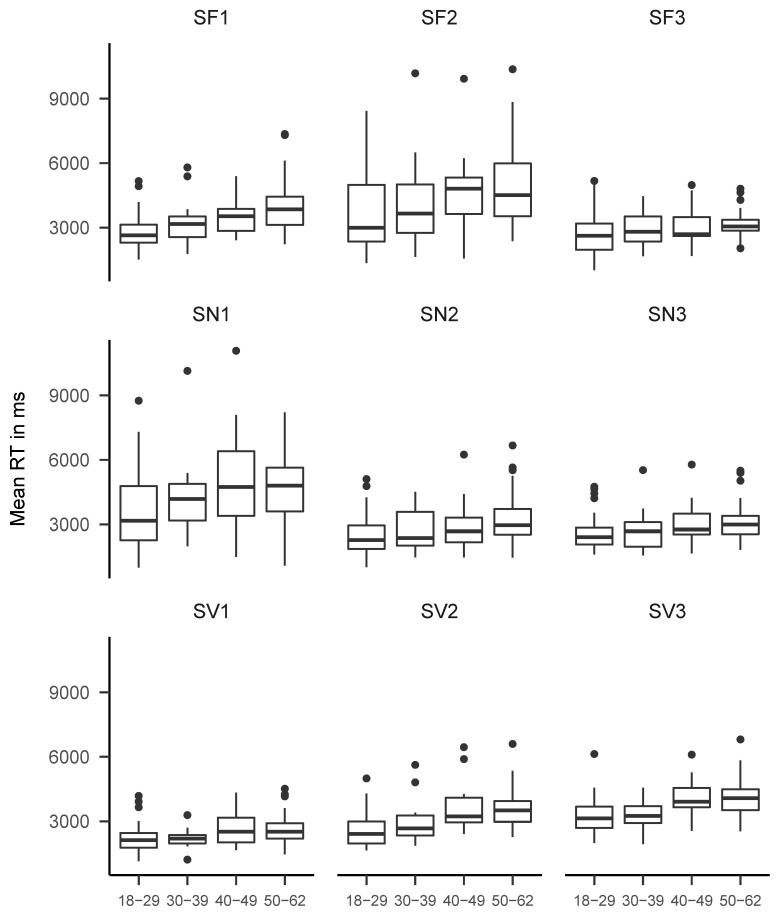
Boxplots of mean response times for all slow tasks, split by age groups. See Table 1 for an explanation of the task names.

**Figure A3:**
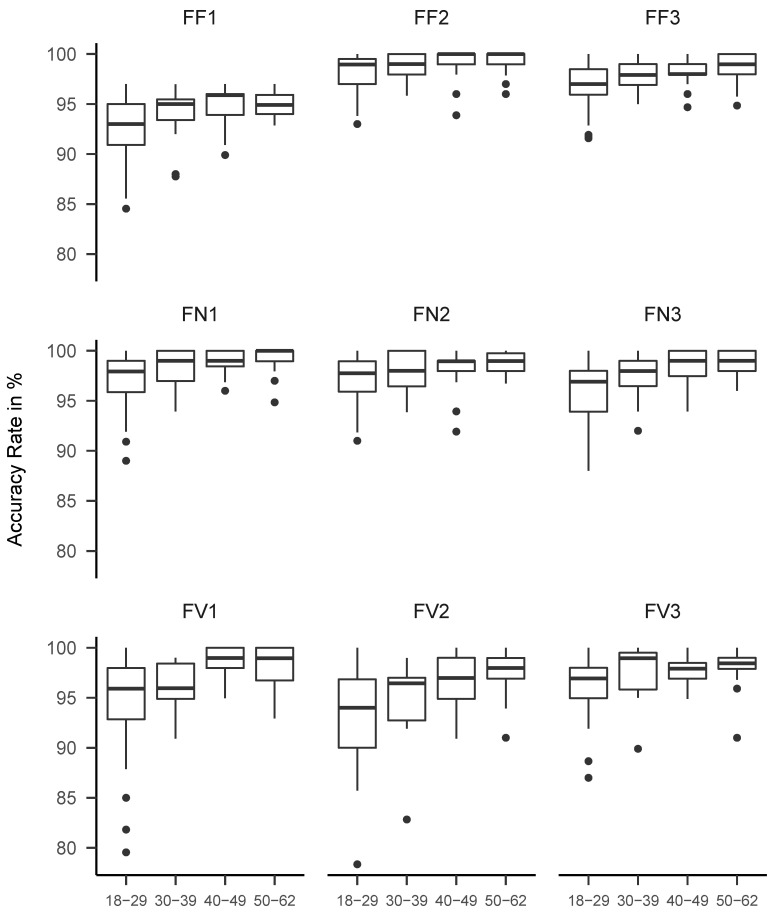
Boxplots of accuracy rates for all fast tasks, split by age groups. See Table 1 for an explanation of the task names.

**Figure A4:**
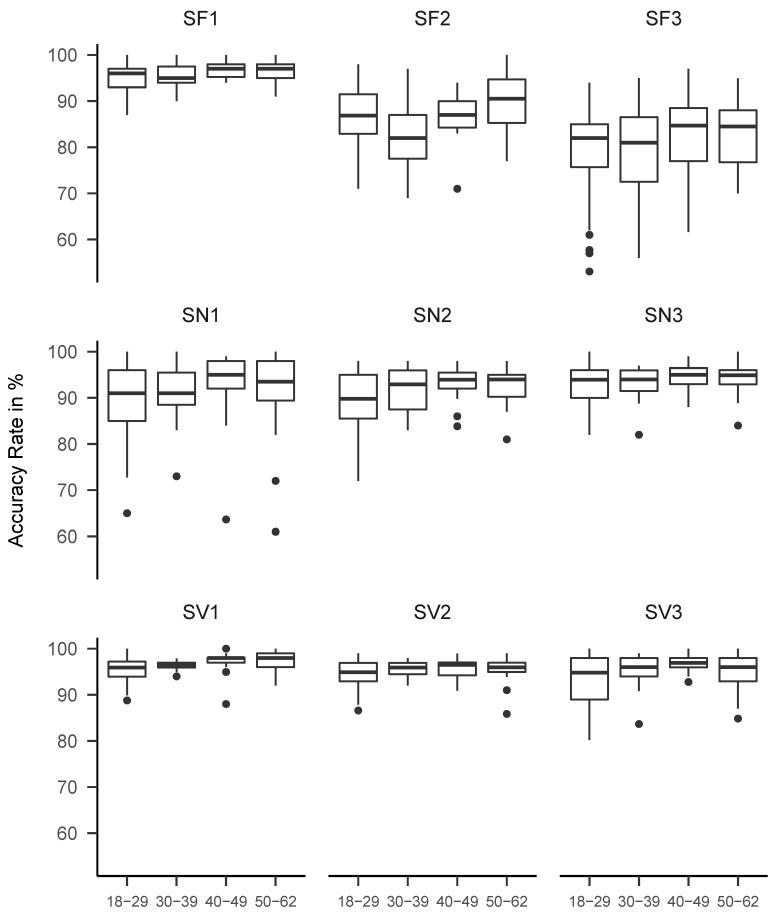
Boxplots of accuracy rates for all slow tasks, split by age groups. See Table 1 for an explanation of the task names.

Figure 5 and Figure 6 show boxplots of the mean response times for the final data set. Mean RT ranged between 527 and 792 ms (*M* = 647 ms) for the fast tasks and between 2380 and 4189 ms (*M* = 3225 ms) for the slow tasks. Table 2 shows descriptive statistics for mean RT, accuracy rate, drift rate, boundary separation, and non-decision time for all tasks. Figure A1, Figure A2, Figure A3 and Figure A4 show boxplots of mean response times and accuracy rates, split by age group.

Table 3 contains the bivariate age correlations of response times, accuracy rates, and all diffusion model parameters for all tasks. Age correlations ranged from −0.34 to 0.25 for drift rate, from 0.11 to 0.49 for boundary separation, and from 0.13 to 0.62 for non-decision time.

In general, there were medium positive age correlations for boundary separation, medium to strong positive age correlations for non-decision time, and no significant correlations between age and drift rate. In addition, it is important to note that there are substantial task-specificities. Some drift rates showed negative age correlations (i.e., the simple area task, the maze task, and the statement task), but in the word category task, older participants had higher drift rates. In addition, for non-decision time and boundary separation, two of the correlations were very low (|*r*| < 0.15) and several values did not reach statistical significance, although the overall trend was clear.

**Figure A5:**
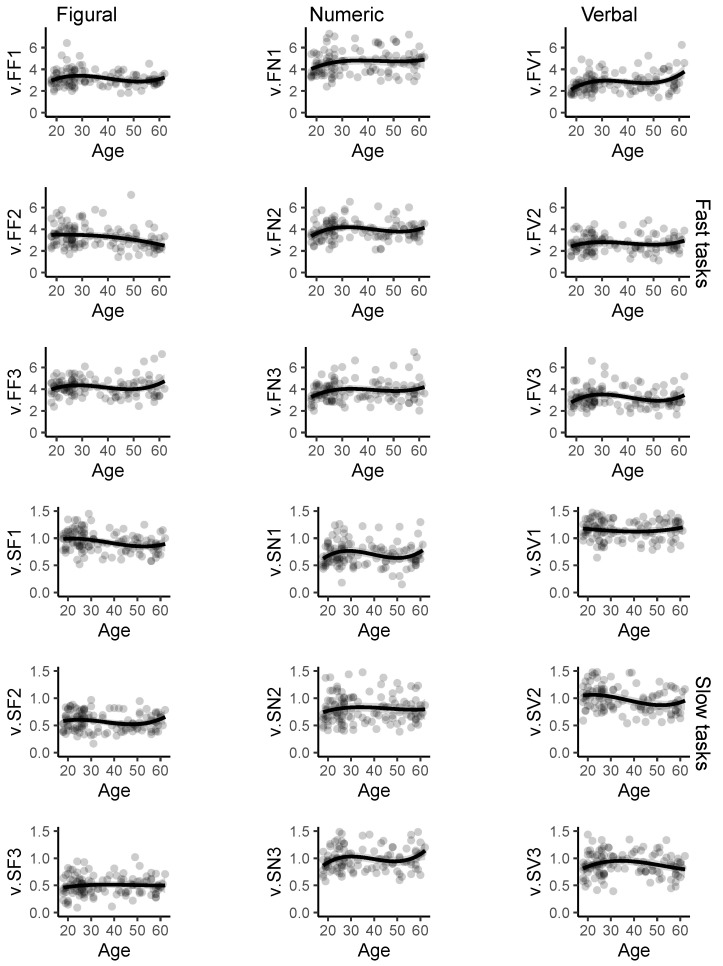
Scatterplots of drift rate vs. age in the 18 different tasks. See Table 1 for task descriptions. The trends show results from a polynomial regression of third degree.

As we did not find linear age correlations for most of the drift rates, we explored the age-drift rate relation by fitting cubic models. Figure A5 in the Appendix A shows the scatter plots across all tasks. Across many tasks, drift rates seemed to rise until about the age of 30, declining thereafter. The rise in drift rate above the age of 60 found in some tasks has to be interpreted with caution, given that we had fewer than five participants of that age group.

Table 4 shows the correlations of response times, accuracy rates, and the diffusion model parameters with general intelligence. All variables were substantially correlated with intelligence, with great variability across tasks. Generally, drift rates in slow tasks showed stronger correlations with intelligence than drift rates in fast tasks.

Table 5 shows the correlations of the aggregated diffusion model parameters, age, and the content-general outcome variables (*g*, processing capacity, psychometric speed, and memory). Table 6 shows the correlations of the aggregated diffusion model parameters, age, and the content-specific outcome variables (figural, numeric, and verbal intelligence).

### 3.2. Mediation Analyses

#### 3.2.1. Mediation Models with *g* as Outcome (Models 1 and 2)

Figure 2 shows the results for the mediation Models 1 and 2 that used *g* as outcome variable and either mean log RT (Model 1) or the diffusion model parameters (Model 2) as mediators. In both models, after introducing the mediating variables, the relation of age and *g* was no longer statistically significant. In Model 1, mean log RT was linked to both age and *g*. The bootstrapped 99.5% confidence interval for the indirect effect of age via mean log RT did not include zero. Mean log RT accounted for 80% of the total effect. In Model 2, age was linked to *t*_0_ and *a*, but not *v*, while *g* was linked to *t*_0_ and *v*, but not *a*. The only indirect effect with a bootstrapped 99.5% confidence interval that did not include zero was for *t*_0_ (non-decision time). The diffusion model parameters accounted for 59% of the total effect.

#### 3.2.2. Mediation Models with Process Domains as Outcomes (Models 3–5)

First, we checked whether mean logarithmized RTs mediated the relation of age and the respective outcome scores. This was the case for all three outcome measures. Accordingly, in the next step, the diffusion model parameters were examined as mediators of the link between age and the intelligence process domains. Figure 3 shows the results for the Models 3–5. In these mediation models, the intelligence process domains processing capacity (Model 3), psychometric speed (Model 4), and memory (Model 5) were used as outcomes, respectively. In all three models, the correlations of age and the intelligence process domains were no longer statistically significant after introducing the mediating variables. In Model 3, processing capacity was linked only to drift rate, but not to boundary separation and non-decision time. Here, all bootstrapped 99.5% confidence intervals of the mediation effects included zero. Still, the diffusion model parameters accounted for 55% of the total effect on processing capacity. In Model 4, psychometric speed was linked to *t*_0_ and *v*, but not to *a*. The only indirect effect with a bootstrapped 99.5% confidence interval that did not include zero was observed for *t*_0_. The diffusion model parameters accounted for 66% of the total effect on psychometric speed. In Model 5, memory was linked to *t*_0_ and *v*, but not to *a*. The only indirect effect with a bootstrapped 99.5% confidence interval that did not include zero was again *t*_0_. The diffusion model parameters accounted for 56% of the total effect on memory.

#### 3.2.3. Mediation Models with Content Domain Scores as Outcomes (Models 6–8)

First, we checked whether domain-specific mean logarithmized mediated the relation of age and the respective outcome scores. This was the case for all three outcome measures. Accordingly, in the next step, the content domain specific diffusion model parameters were examined as mediators of the link between age and the intelligence content domain scores. Figure 4 shows the results for the Models 6–8. In these mediation models, the figural (Model 6), numerical (Model 7), and verbal (Model 8) intelligence scores were used as outcomes, respectively. For figural intelligence (Model 6), the age correlation remained significant even after introducing the mediators. Figural intelligence was linked only to drift rate, but not to boundary separation and non-decision time. Age was correlated only to figural non-decision time and figural boundary separation, but not to figural drift rate. All bootstrapped 99.5% confidence intervals of the mediation effects included zero. The diffusion model parameters accounted for 30% of the total effect on figural intelligence. In the verbal and numerical models, the correlation of age and the intelligence scores was no longer statistically significant after introducing the mediating variables. In Model 7, numerical intelligence was linked to numerical *t*_0_, *a*, and *v*. Age was correlated only to numerical non-decision time and numerical boundary separation, but not to numerical drift rate. The only indirect effect with a bootstrapped 99.5% confidence interval that did not include zero was for *t*_0_. The diffusion model parameters accounted for 96% of the total effect on numerical intelligence. In Model 8, verbal intelligence was linked to verbal *t*_0_ and *v*, but not to *a*. Age was correlated only to verbal non-decision time and verbal boundary separation, but not to verbal drift rate. The only indirect effect with a bootstrapped 99.5% confidence interval that did not include zero was for *t*_0_. The diffusion model parameters accounted for 59% of the total effect on verbal intelligence.

## 4. Discussion

Results from several studies show that response times from elementary cognitive tasks substantially mediate the relation of age and cognitive abilities (Finkel et al. 2007; Salthouse 1996), suggesting that age differences in intelligence might be (partly) based on age differences in processing speed. However, response times are not process-pure measures, as they reflect not only the speed of information processing, but also—for example—speed-accuracy trade-offs or the time needed for sensory encoding and motor response execution. The diffusion model (Ratcliff 1978) provides separate estimates for these different components of the decision process. A previous study demonstrated that not processing speed but non-decision time mediates the relation of performance in elementary cognitive tasks and general intelligence (Schubert et al. 2020). The present study builds upon this finding and aims at testing which components of information processing mediate the link of age and decline in a range of intelligence content domains and intelligence process domains.

For the present study, we used a wide range of response time tasks across different content domains. In previous studies on the age effects in diffusion model parameters, only a limited number of tasks have been examined simultaneously so that it was not possible to examine effects of content domain systematically (e.g., Ratcliff et al. 2001, 2004, 2010; McKoon and Ratcliff 2012; Ratcliff 2008). Of the 18 response time tasks employed in our study, six belonged to the figural, numeric, and verbal domain, respectively. Furthermore, half of the tasks were based on fast decisions, while the other half were more complex tasks and required much longer processing times. As outcomes, we did not only examine *g*, but also different intelligence scores (processing capacity, psychometric speed, and memory). Thus, we could examine the generalizability of the non-decision time mediation reported by Schubert et al. (2020) across content domains, task complexities, and intelligence process domains.

An additional important difference between our study and most previous studies on age differences in diffusion model parameters is that we studied a broad age range from 18 to 62 years, whereas most previous studies had compared only two age groups, college age people and old adults (65+ years old). In contrast, our sample included 66 persons from mid-adulthood, aged 30–60 years, an age group that is understudied in diffusion model analyses so far. Previous studies found compelling evidence for an age-related increase of boundary separations and non-decision times (for a meta-analysis of age-effects on diffusion-model parameters, see Theisen et al. 2020). That is, elder adults are more cautious decision-makers and they are slower in encoding and/or motoric response execution. In our study, we could assess whether age differences found for the group comparisons map onto linear age correlations across a wider range of adulthood.

For most of the 18 employed RT tasks, we found strong age correlations of mean logarithmized response times reflecting slower responses for elder participants. Correlations between age and RT tended to be higher for fast than for slow tasks, and among the slow tasks correlations were more heterogeneous. This last finding might reflect greater task complexity of the slow tasks, which might lead to greater between-task variability in the cognitive processes and in the abilities required for solving the tasks, thus resulting in different age correlations.

Non-decision times—as estimated by the diffusion model—showed medium to strong correlations with age for most tasks. This implies that older participants take longer for encoding and/or motor processes. As expected, age was also related to boundary separation, though to a smaller degree. This implies that for most tasks, older participants tend to apply more conservative decision criteria, indicating that they gather more information before making a decision. These results are perfectly in line with results from the recent meta-analysis by Theisen et al. (2020), although it should be noted that the meta-analysis compared young adults and old adults, while our study focused on young- and mid-adulthood. Our pattern of results suggests a continuous developmental increase in cautiousness—elder people get more conservative and take more time for encoding and motor execution. Of course, our cross-sectional design does not allow for a direct test of this hypothesis.

Regarding speed of information processing (drift rate), we found no age correlations for most of the tasks. We also did not find a clear pattern of differences in the age-drift correlation between the three different content domains or for fast vs. complex tasks. Younger people had higher drift rates in some, but not in all figural tasks. Regarding drift rates in verbal tasks, we had expected older people to have an advantage, as Theisen et al. (2020) report that task content moderates the age effects on drift, with an age-related increase for lexical decision tasks. In our sample, older people had higher drift rates in one verbal task (the noun-adjective task, but not in the lexical decision task). For the other verbal tasks, we found no correlations between drift rate and age, except for a negative age correlation in the statement task. In this regard, our findings regarding the drift rate are not in line with the effects reported by Theisen et al. (2020). In exploratory analyses, we fitted cubic models to examine a possible nonlinear relationship of age and drift. Interestingly, for drift rates from many tasks as well as for the composite drift rate across tasks, we found evidence for a positive age trend from age 18 until about the age of 30. After that, drift rates showed a linear negative age trend until about the age of 60. These findings suggest that many previous studies might not have found significant age effects in drift rate because they compared very young people (i.e., in their early twenties) to old adults (65+ years). A similar interpretation has also been proposed for findings on different cognitive abilities like, for example, working memory (Hartshorne and Germine 2015). Indeed, when excluding our youngest participants (i.e., people aged 18–29), we found small to medium negative age correlations for drift rates across several tasks—most of them were fast tasks. As excluding these young adults made our sample considerably smaller, the findings should be interpreted with caution. Still, future studies might be well advised to include people in the mid-adult age range to get a clearer picture on where the turning point in the development of processing speed lies. Ideally, one could study the trends longitudinally, measuring participants repeatedly from college age into middle or even old adulthood.

Our main research question was whether diffusion model parameters could explain age differences in intelligence. First of all, we replicated the finding that logarithmized mean response times fully mediated the correlation between age and general intelligence (Finkel et al. 2007; Salthouse 1996). The models using the diffusion model parameters as mediators of the age effect on intelligence showed a robust indirect effect for non-decision time, indicating that the age related decline in intelligence test scores is mediated by the duration of encoding and/or motor processes. Drift rates were clearly linked to *g*, but not to age, and thus did not show a significant indirect effect. Boundary separation was linked to age, but not to *g*, also leading to an insignificant indirect effect. The three diffusion model parameters jointly fully mediated the relation between age and *g*.

These findings replicated across most of the analyses using the process domain scores (processing capacity, psychometric speed, memory) and the content domain scores (figural, numerical, verbal) as outcomes. Drift rates were linked to the intelligence outcomes, but not to age. The only exception for the latter was in the figural content domain, where figural drift rates showed a small negative correlation to age and the indirect effect via drift rate accordingly approached statistical significance. Boundary separation was not linked to the intelligence outcomes, except for numerical intelligence, where numerical boundary separation showed a small negative correlation to numerical intelligence and the indirect effect via boundary separation accordingly approached statistical significance. Finally, non-decision time was linked to both age and the intelligence outcomes in all cases except processing capacity and figural intelligence, which showed no significant correlations to the respective non-decision times. These findings suggest that age differences in processing capacity and figural intelligence are not based on age differences in any of the diffusion model parameters.

The correlation of intelligence with non-decision time was particularly strong for the psychometric speed scores, indicating that this intelligence scale is strongly influenced by speed in sensory encoding and/or motor response execution, but not necessarily by speed of information processing, as drift rates showed no correlation. Schubert et al. (2020) offered two different possible explanations for the mediation of the age to *g* relation through non-decision time. On the one hand, age-related variation in non-decision time might reflect age-related variation in anterior brain areas associated both with response preparation and other higher-order processes, implying that the non-decision time mediation generalizes across process domains. On the other hand, the indirect effect might be overestimated, as performance in intelligence test tasks involves a component of motoric speed. The degree to which motor speed is involved differs between intelligence tests—the psychometric speed tasks of the BIS that rely extensively on quick hand-writing, should in this case be strongly related to non-decision time. Our finding that non-decision time was particularly closely related to scores in the psychometric speed tasks of the BIS test could thus be viewed as support to this latter notion, implying that speed of motor response execution plays an important role in determining the relationship of non-decision time, age, and intelligence test scores. On the contrary, reasoning tasks that are closer to a power test and rely less on time pressure, should show strong relations to processing speed, and be less correlated to non-decision time. This is exactly the pattern we find in our data, with the processing capacity tasks being the closest to a power test among the BIS tasks. These results bring up the question of whether the mediation of age differences in *g* scores via non-decision time truly informs us about intelligence, or is partly an artifact of the speeded intelligence test tasks. Using a power test without any time limit as an outcome might be the next step to further investigate this issue.

One important issue when studying age differences in cognition is whether these differences and developmental patterns are general or domain-specific. Given our finding that non-decision times mediated age differences in intelligence for the verbal and numerical content domains, we conducted additional analyses to investigate whether it was the domain-general or domain-specific parts of variance in non-decision time that accounted for the mediation. To this end, we estimated a simple structural equation model, using the three non-decision time values from figural, numerical, and verbal non-decision times as indicators of a general non-decision time factor. We then used this general non-decision time and domain-specific non-decision time as mediators of the relationship between age and domain-specific intelligence. It turned out that it was the general non-decision time factor, but not the domain-specific non-decision time residuals that accounted for the indirect effect, both in the numerical and in the verbal domain. The domain-specific non-decision time residuals were not related to age. This suggests that the processes eliciting age differences in non-decision time generalize across domains.

Taken together, our findings suggest that the often reported age-related slowing in response time tasks, which mediates the relationship between age and a wide range of cognitive abilities, can mostly be attributed to the fact that older people take longer for non-decisional processes. This finding proved to be robust across a range of cognitive ability outcomes including general intelligence and memory, with the exception of processing capacity and figural intelligence.

It is important to note that all our outcome tasks were speeded and our findings might therefore be partly overstating the relationship between non-decision time and general cognitive ability. In the least speeded intelligence tasks—namely, those assessing processing capacity and thus probably most closely reflective of reasoning ability—non-decision time was not a mediator, but neither was processing speed (i.e., drift rate). In this sense, all our findings contradict the idea that a decline in processing speed is the basis of cognitive decline in general. Our results are more easily reconcilable with the assumption of a “common cause” (Christensen et al. 2001) that is related to decline in a wide range of cognitive abilities, including response times—the age relationship of the latter being, according to our analyses, in large parts defined by the time taken for motor processes. At the same time, the variability in correlations of non-decision time with age and IQ across tasks implies the importance of domain-specific factors. The literature on the relation between age differences in cognition and in brain structure suggests correlated change, but findings greatly differ regarding the strength of this relationship (for a review, see Oschwald et al. 2019). Findings on processing speed are also inconclusive in this regard. According to the Scaffolding Theory of Aging and Cognition (STAC-r; Reuter-Lorenz and Park 2014), people employ different compensatory scaffolding techniques (e.g., strategy use, activation of additional brain networks) to counter the detrimental effects of age-related alterations in brain structure. Differences in coping abilities might thus influence the relations between brain structure and cognitive abilities. Regarding the diffusion model parameters, drift rates might reflect a type of processing speed that is open to compensatory scaffolding techniques and thus relatively stable across a large part of the life-span, while the more basic processes contributing to non-decision times might be less malleable and thus show clearer age correlations.

One important additional finding is the great variability of age correlations for the diffusion model parameters across the 18 tasks employed in the present study. For drift rates, no age correlations were found in most of the tasks. However, in two figural tasks, elder people showed lower drift rates. At the same time, in one verbal task (the noun-adjective task), elder persons had higher drift rates. These findings underline the importance of using a wider variety of tasks when studying age differences in diffusion model parameters. Had we only used one or two tasks, the general picture might have looked quite different, maybe implying age-related decline in drift rates. The same holds true for the age correlations in boundary separation and non-decision time. Even though the general picture is quite clear in both cases—medium to large age correlations—there are several tasks where either boundary separation, non-decision time, or both parameters are not related to age. Thus, the wide range of response time tasks employed proves to be an important strength of this study.

### 4.1. Limitations

For diffusion modeling, the number of trials per task and participant was rather low. We decided to employ a wide range of tasks instead of just a few tasks with high numbers of trials. Simulation studies suggest that the diffusion model yields adequate estimates for 100 trials (Lerche et al. 2017). We also examined model fit, which was good for all tasks in our study.

A second important limitation of our study is that also the sample size is limited. This has implications for the modeling approach employed. One could argue that aggregating parameters across tasks simply by computing the mean of the standardized values is an oversimplification of the structure of drift rates, boundary separation values, non-decision times, and response times. The procedure implies the assumption of parallel measurement, that is, the presumption that all items contribute equally and fully to a common latent factor. This is a strong assumption that cannot be tested in the modeling approach we used. Unfortunately, investigating the mediations through latent variable structural equation modeling including all task-specific diffusion model parameters, such as in the approach used by Schubert et al. (2020), was impossible due to our restricted sample size. To address this issue, we estimated principal component analyses, separately for each of the diffusion model parameters and mean log RTs (across all 18 tasks, and separately for each content domain). In each principal component analysis, we assumed one general factor, to mirror the factor structure from our main analyses. We then extracted factor scores and used these as mediators in the mediation models (Models 1–8). This did not alter the interpretation of any of the results. In fact, factor scores were highly correlated (often *r* = 0.99) to the means of standardized task scores. This implies that our simple aggregation procedure (means of standardized values across tasks) is justified. At the same time, the range of age and IQ correlations across tasks hints at task-specific aspects and/or sub-factors.

We also estimated separate structural equation mediation models for each diffusion model parameter and each content domain, for example, a mediation model with age as predictor, numerical intelligence as outcome, and numerical non-decision time as the only mediator—the latter being a latent factor linked to non-decision times in all numerical tasks. Though several of these models suffered from inadequate model fit and results from these models must thus be interpreted with caution, these additional analyses did not indicate a different pattern of results than our main analyses. All these analyses can be replicated using the scripts on the paper’s OSF page (https://osf.io/xpbwe).

It is critical to note that mediation models cannot provide a test of causal relations. In fact, one could think of a number of different models that would show identical model fit, but assume a completely different causal relationship of the variables. While the models tested in our study are based in theory, there is no way to tell if they reflect the “true” causal relationships between age, the diffusion model parameters, and cognitive abilities. Another important limitation is the fact that age and cohort effects are confounded in our study—a problem that could only be fully remedied by following several different cohorts longitudinally.

Future studies might shed light on the question what accounts for the age differences in processing capacity and figural intelligence, as they were unrelated to non-decision time in our sample. These studies should include measures of working memory capacity and executive functions, as well as neuro-cognitive data, to disentangle the non-speed related processes that might account for age differences in cognition.

A final limitation of our study is the fact that we did not include people older than 62 years. Thus, we cannot examine the developmental patterns that occur in old age. Ratcliff et al. (2006) found significant differences in diffusion model parameters between people aged 60–74 and those older than that. In comparison with participants aged 60–74, the eldest participants (aged 75–85) had more conservative decision criteria, longer non-decision times, and lower drift rates, though all these findings differed between tasks. It would be highly interesting to expand the mediation analyses to this age group to assess whether the correlational patterns are qualitatively different here.

### 4.2. Conclusions

Cognitive slow-down is thought to contribute to the age-related decline found for a wide range of cognitive abilities, including general intelligence. We investigated the relationships between age, three main diffusion model parameters calculated from 18 different response time tasks, and different measures of intelligence. Older people in our sample (ranging from young adulthood to the beginning of old age) used more conservative decision criteria and needed more time for extra-decisional processes, but no linear age effect was found for processing speed. Individual differences in non-decision times fully mediated the relation between age and intelligence for most measures of intelligence. Only scores of processing capacity and figural intelligence did not show a significant relationship to non-decision time. Our findings support the account that, already in mid-adulthood, age differences in intelligence test scores are based on age differences in non-decisional processes, in particular motor execution time.

## Figures and Tables

**Table 1:** Overview of the 3 (domain: figural vs. numeric vs. verbal) × 2 (speed: fast vs. slow) × 3 (number of tasks) = 18 RT tasks.

	Fast	Slow
	FF1: dot-rectangle task (1.9)	SF1: maze task (2.1)
Figural	FF2: simple area task (2.4)	SF2: complex area task (1.6)
	FF3: polygon task (1.3)	SF3: pie task (2.7)
	FN1: number discrimination task (2.2)	SN1: mean value computation task (1.8)
Numeric	FN2: odd-even task (1.5)	SN2: equation task (2.5)
	FN3: simple inequation task (2.8)	SN3: complex inequation task (1.2)
	FV1: word category task (2.6)	SV1: grammar task (1.4)
Verbal	FV2: lexical decision task (1.1)	SV2: statement task (2.3)
	FV3: animacy task (1.7)	SV3: semantic category task (2.9)

*Note*. The first letter indicates the task complexity (F = fast, S = slow); the second letter denotes the domain (N = numeric, V = verbal, F = figural). The numbers in parentheses indicate the time point of assessment (session and number in sequence).

**Table 2:** Means (*M*) and Standard Deviations (*SD*) of Mean RTs in ms, Accuracy Rates (in %), and the Diffusion Model Parameters for all 18 Tasks.

Task	*M*_RT_	*SD*_RT_	*M*_Acc._	*SD*_Acc._	*M_v_*	*SD_v_*	*M_a_*	*SD_a_*	*M_t_*_0_	*SD_t_*_0_
FF1	560	96	93.65	2.88	3.16	0.73	0.91	0.21	0.42	0.07
FF2	620	176	98.68	1.60	3.26	1.02	1.53	0.53	0.36	0.07
FF3	551	96	97.71	1.90	4.27	0.96	1.16	0.61	0.41	0.06
FN1	527	78	98.03	2.26	4.97	1.82	1.47	1.31	0.39	0.07
FN2	590	107	97.68	2.03	3.95	0.97	1.20	0.51	0.43	0.06
FN3	670	135	97.17	2.74	3.97	1.39	1.36	1.03	0.50	0.10
FV1	792	164	96.22	3.76	2.81	0.88	1.52	0.73	0.51	0.08
FV2	781	162	95.11	3.97	2.68	0.78	1.33	0.44	0.53	0.07
FV3	737	124	97.18	2.41	3.21	0.89	1.35	0.55	0.52	0.07
SF1	3234	1091	95.53	2.91	0.94	0.20	3.75	1.44	1.29	0.49
SF2	4189	2009	86.69	6.50	0.58	0.17	3.71	1.37	1.48	0.92
SF3	2856	906	80.47	9.10	0.50	0.18	3.06	0.81	0.91	0.40
SN1	4168	1904	90.76	8.11	0.70	0.22	4.00	1.53	1.63	1.21
SN2	2761	1098	91.16	5.48	0.80	0.25	3.25	0.92	0.84	0.31
SN3	2805	885	93.51	3.71	1.08	0.33	2.85	0.92	1.50	0.42
SV1	2380	709	96.36	2.39	1.17	0.20	3.08	0.84	1.09	0.35
SV2	3030	1002	95.11	2.61	1.03	0.29	3.19	0.87	1.45	0.42
SV3	3600	895	94.24	4.77	0.90	0.23	3.69	1.23	1.64	0.41

*Note*. See Table 1 for an explanation of the task names. Diffusion Model parameters: *v*: drift rate; *a*: boundary separation; *t*_0_: non-decision time.

**Table 3:** Age correlations of RTs, accuracy rates, and diffusion model parameters for all 18 RT tasks.

Task	Mean RT	Mean log. RT	Accuracy Rate	Drift Rate	Boundary Sep.	Non-Decision Time
FF1	0.64 **	0.66 **	0.41 **	−0.16	0.43 **	0.62 **
FF2	0.54 **	0.57 **	0.27 *	−0.29 *	0.37 **	0.50 **
FF3	0.56 **	0.60 **	0.37 **	0.01	0.38 **	0.49 **
FN1	0.61 **	0.62 **	0.43 **	0.02	0.16	0.37 **
FN2	0.32 **	0.37 **	0.39 **	0.01	0.25	0.35 **
FN3	0.59 **	0.60 **	0.50 **	0.09	0.34 **	0.40 **
FV1	0.28 *	0.32 **	0.46 **	0.25	0.36 **	0.25
FV2	0.37 **	0.40 **	0.48 **	0.02	0.49 **	0.17
FV3	0.46 **	0.48 **	0.34 **	−0.07	0.21	0.44 **
SF1	0.50 **	0.51 **	0.28 *	−0.31 **	0.33 **	0.25 *
SF2	0.25	0.32 **	0.23	−0.08	0.22	0.28 *
SF3	0.24	0.31 **	0.18	0.05	0.22	0.19
SN1	0.26	0.27 *	0.17	−0.05	0.22	0.13
SN2	0.25 *	0.28 *	0.29 *	0.01	0.25	0.29 *
SN3	0.25 *	0.30 **	0.20	0.02	0.11	0.42 **
SV1	0.31 **	0.32 **	0.35 **	0.00	0.25	0.31 **
SV2	0.48 **	0.51 **	0.19	−0.34 **	0.45 **	0.32 **
SV3	0.45 **	0.47 **	0.24	−0.09	0.32 **	0.30 **

*Note*. See Table 1 for an explanation of the task names. * *p* < 0.005, ** *p* < 0.001.

**Table 4:** IQ correlations of RTs, accuracy rates, and diffusion model parameters for all 18 RT tasks.

Task	Mean RT	Mean log. RT	Accuracy Rate	Drift Rate	Boundary Sep.	Non-Decision Time
FF1	−0.46 **	−0.46 **	−0.33 **	0.13	−0.34 **	−0.44 **
FF2	−0.46 **	−0.44 **	−0.19	0.32 **	−0.35 **	−0.25
FF3	−0.62 **	−0.63 **	−0.21	0.25	−0.29 *	−0.45 **
FN1	−0.57 **	−0.57 **	−0.13	0.18	−0.07	−0.36 **
FN2	−0.60 **	−0.64 **	−0.28 *	0.33 **	−0.33 **	−0.48 **
FN3	−0.67 **	−0.69 **	−0.27 *	0.15	−0.27 *	−0.48 **
FV1	−0.48 **	−0.50 **	−0.12	0.21	−0.28 *	−0.29 *
FV2	−0.49 **	−0.50 **	−0.12	0.22	−0.38 **	−0.34 **
FV3	−0.51 **	−0.53 **	−0.08	0.32 **	−0.18	−0.41 **
SF1	−0.54 **	−0.54 **	−0.04	0.46 **	−0.38 **	−0.21
SF2	−0.35 **	−0.40 **	0.03	0.37 **	−0.28 *	−0.21
SF3	−0.22	−0.24	0.25	0.34 **	−0.07	−0.23
SN1	−0.26 *	−0.23	0.24	0.41 **	0.00	−0.25
SN2	−0.66 **	−0.71 **	0.10	0.60 **	−0.55 **	−0.44 **
SN3	−0.67 **	−0.72 **	−0.06	0.44 **	−0.52 **	−0.49 **
SV1	−0.54 **	−0.55 **	−0.20	0.29 *	−0.34 **	−0.51 **
SV2	−0.56 **	−0.57 **	−0.02	0.42 **	−0.45 **	−0.42 **
SV3	−0.62 **	−0.64 **	0.01	0.42 **	−0.41 **	−0.25

*Note*. See Table 1 for an explanation of the task names. * *p* < 0.005, ** *p* < 0.001.

**Table 5:** Correlations of all the variables used for the general mediation analyses.

	1	2	3	4	5	6	7	8
1—Age								
2—*g*	−0.47 **							
3—Processing Cap.	−0.37 **	0.91 **						
4—Psy. Speed	−0.44 **	0.78 **	0.55 **					
5—Memory	−0.39 **	0.75 **	0.55 **	0.48 **				
6—mean log RT	0.58 **	−0.70 **	−0.59 **	−0.63 **	−0.55 **			
7—*t*_0_	0.57 **	−0.60 **	−0.46 **	−0.57 **	−0.51 **	0.78 **		
8—*a*	0.50 **	−0.51 **	−0.44 **	−0.47 **	−0.38 **	0.89 **	0.50 **	
9—*v*	−0.08	0.60 **	0.57 **	0.40 **	0.47 **	−0.52 **	−0.23	−0.34 **

*Note*. ** *p* < 0.001.

**Table 6:** Correlation matrix of the variables used for the content-domain specific mediation analyses.

	1	2	3	4	5	6	7	8	9	10	11	12
1—Age												
2—Verbal IQ	−0.41 **											
3—Figural IQ	−0.52 **	0.53 **										
4—Numerical IQ	−0.26 *	0.56 **	0.53 **									
5—*t*_0_ Verbal	0.44 **	−0.59 **	−0.31 **	−0.43 **								
6—*t*_0_ Figural	0.60 **	−0.40 **	−0.40 **	−0.33 **	0.70 **							
7—*t*_0_ Numerical	0.50 **	−0.54 **	−0.41 **	−0.59 **	0.66 **	0.72 **						
8—*v* Verbal	−0.04	0.53 **	0.24	0.37 **	−0.28 *	−0.08	−0.25 *					
9—*v* Figural	−0.21	0.38 **	0.52 **	0.38 **	−0.07	−0.16	−0.27 *	0.49 **				
10—*v* Numerical	0.03	0.39 **	0.27 *	0.60 **	−0.10	0.01	−0.29 *	0.50 **	0.53 **			
11—*a* Verbal	0.49 **	−0.52 **	−0.38 **	−0.35 **	0.43 **	0.51 **	0.48 **	−0.41**	−0.33 **	−0.15		
12—*a* Figural	0.50 **	−0.47 **	−0.38 **	−0.25	0.35 **	0.36 **	0.39 **	−0.24	−0.39 **	−0.13	0.78 **	
13—*a* Numerical	0.36 **	−0.47 **	−0.36 **	−0.35 **	0.42 **	0.43 **	0.29 *	−0.25	−0.34 **	−0.09	0.73 **	0.73 **

*Note*. * *p* < 0.005, ** *p* < 0.001.
